# Fishnet‐Like, Nitrogen‐Doped Carbon Films Directly Anchored on Carbon Cloths as Binder‐Free Electrodes for High‐Performance Supercapacitor

**DOI:** 10.1002/gch2.201900086

**Published:** 2020-01-08

**Authors:** Jing Wu, Liming Xu, Weiqiang Zhou, Fengxing Jiang, Peipei Liu, Hui Zhang, Qinglin Jiang, Jingkun Xu

**Affiliations:** ^1^ Jiangxi Engineering Laboratory of Waterborne Coatings Jiangxi Science and Technology Normal University Nanchang 330013 China; ^2^ College of Chemistry and Molecular Engineering Qingdao University of Science & Technology Qingdao 266042 China

**Keywords:** binder‐free flexible carbon films, in situ N‐doping, polyindole, porous carbons, supercapacitors

## Abstract

The low specific capacitance and energy density of carbon electrode has extremely limited the wide application of supercapacitors. For developing a high‐performance carbon electrode using a simple and effective method, a fishnet‐like, N‐doped porous carbon (FNPC) film is prepared by calcining the KOH‐activated polyindole precoated on carbon cloths. The FNPC film is tightly anchored on carbon cloths without any binder. The FNPC film with 3.8 at% N content exhibits a fairly high specific capacitance of 416 F g^−1^ at 1.0 A g^−1^. Moreover, the assembled button‐type cell with two FNPC film electrodes shows a high energy density of 16.4 Wh kg^−1^, a high power density of 67.4 kW kg^−1^, and long‐term cyclic stability of 92% of the initial capacitance after 10 000 cycles at 10 A g^−1^. The high performances mainly came from the integration of pseudocapacitance and electrical double‐layer capacitance behavior, wettability, fishnet‐like nanostructure, as well as the low interfacial resistivity. This strategy provides a practical, uncomplicated, and low‐cost design of binder‐free flexible carbon materials electrode for high‐performance supercapacitors.

## Graphical abstract

Fishnet‐like, hydrophilic, N‐doped porous carbon film is prepared through the calcination of polyindole on carbon cloths. The film exhibits a high specific capacitance of 416 F g^−1^ at 1.0 A g^−1^. The assembled supercapacitor has a high energy density of 16.4 Wh kg^−1^, maximum power density of 67.4 kW kg^−1^, and excellent long‐term cyclic stability.

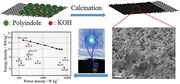

## 1 Introduction

In the past decades, supercapacitors, as one of the most promising energy storage devices, have attracted tremendous attention in both academic studies and industrial applications due to their higher power density, longer cycle stability, faster charging and discharging rates and more reliable security than batteries.^1^, ^2^ The rapid development of supercapacitors is closely related to electrode materials. Among various electrode materials, carbon materials have been recognized as commercially viable products for supercapacitors owing to their outstanding advantages such as large specific surface areas, high electronic conductivity and excellent chemical stability.^3^ However, the low specific capacitance (100–200 F g^−1^) and low energy density (normally lower than 10 Wh kg^−1^) of carbon materials count against the wide application of supercapacitors.^4^, ^5^ Therefore, many efforts have been done to enhance the capacitance performance of the carbon materials that summarized as follows: i) microstructure control of carbon materials to increase the surface area and tune the pore size distribution;^6^, ^7^ ii) the introduction of heteroatoms (especially nitrogen atoms) into carbon materials to provide an extra pseudocapacitance behavior;^8^, ^9^, ^10^ iii) composite fabrication by combining carbon materials with pseudocapacitance materials such as transition metal oxides and/or conducting polymers.^11^, ^12^, ^13^ The composite of carbon and pseudocapacitance materials can show relative high specific capacitance, but their cycle life and rate capability are inferior due to the intrinsic low electronic conductivity and instability of pseudocapacitance materials.^14^, ^15^

N‐doped porous carbon materials, covering simultaneously the distinguishing features of the microstructure and heteroatoms, not only can improve the specific capacitance of carbon electrode but also retain its intrinsic excellent cycling stability. Up to now, two methods have been used to introduce heteroatoms into carbon materials, including post‐treatment doping and in situ doping. Post‐treatment doping is to introduce heteroatoms into carbon materials such as carbon nanotube and graphene using ammonia gas or urea as nitrogen source.^16^, ^17^, ^18^ However, this method is difficult to prepare homo‐dispersed N‐doped carbon due to the doping reaction mainly occurred on the surface of carbon materials rather than inside.^5^ Furthermore, the high cost of carbon nanotube and graphene limits the widespread commercialization for supercapacitors.^19^ Alternatively, the in situ doping is a direct carbonization procedure of N‐containing polymers such as polydiaminonaphthalene (PDAN), poly(acrylonitrile) (PAN), polyaniline (PANI), or polypyrrole (PPy). The N‐containing polymers can provide C and N sources during the direct carbonization, which are attractive to prepare the homo‐dispersed N‐doped porous carbon materials without any templates.^20^ In comparison with pristine carbon‐based materials, the specific capacitance of the reported N‐doped porous carbon materials prepared by the in situ doping method can be enhanced effectively. For example, Zhu et al. prepared hierarchical N‐doped porous carbon derived from PANI nanotubes using direct carbonization, as‐prepared N‐doped carbon material showed a specific capacitance of 366 F g^−1^ at 0.1 A g^−1^.^20^ Yuan et al. reported that the N‐doped carbon nanowires prepared from the direct carbonization of PANI nanowires showed a specific capacitance of 327 F g^−1^ at 0.1 A g^−1^.^21^ Wei et al. fabricated a polypyrrole‐derived activated carbons, which had a specific capacitance of 290 F g^−1^ at 0.1 A g^−1^.^22^ However, their rate capabilities were about 60% in the range of 0.1–10 A g^−1^. In these examples, the nonconducting polymer binders were used to bond the N‐doped porous carbon materials on the current collector, which inevitably increased the resistance of the electrode, thus reducing the rate capability of the carbon materials. Moreover, the additive of polymer binders may on one hand make the accumulation of carbon materials, on the other hand increase the “dead weight/volume” of electrode, which results in the reduction in the utilization and capacitance contribution of the active electrode materials.

For improving the capacitance performance of the electrode materials, an alternative strategy that the fabrication of the binder‐free carbon electrode has been developed in recent years, such as electrophoretic deposition,^23^ chemical vapor deposition,^24^ and laser direct writing.^25^ These methods were high‐cost, time‐consuming, and complex processes. Different from the above methods, herein, we presented a facile, practical and cost‐effective way to prepare a fishnet‐like hydrophilic N‐doped porous carbon (FNPC) film that tightly anchored on flexible carbon cloths (CC) substrate through the direct calcination of precoating polyindole (PIn) on CC. As a binder‐free electrode, the FNPC film anchored on CC substrate exhibited a fairly high specific capacitance of 416 F g^−1^ at 1.0 A g^−1^. Moreover, the assembled button‐type cell had high energy density of 16.4 Wh kg^−1^, maximum power density of 67.4 kW kg^−1^, and long‐term cyclic stability.

## 2 Results and Discussion

**Figure 1:**
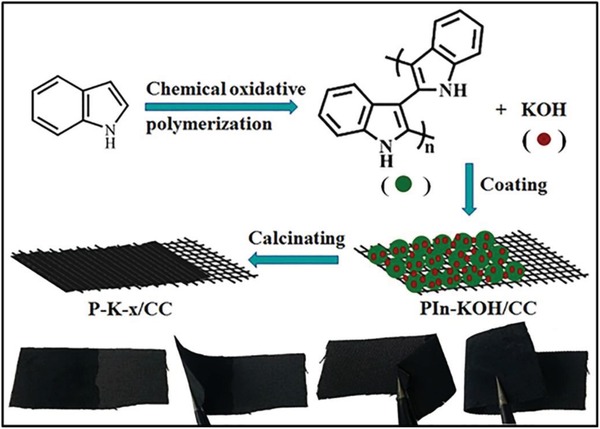
Schematic illustration of preparation of flexible P‐K‐*x*/CC electrode and its twisted states.

To prepare the binder‐free carbon electrodes, the PIn/KOH precursor was firstly coated on CC and then one‐step calcinated to P‐K‐*x* carbon film. As‐prepared P‐K‐*x* carbon film can directly anchored on CC and form a robust flexibility electrode (**Figure** **1**). The resistance between P‐K‐2.5 film and CC was up to 6.0 Ω sq^−1^, close to that of CC (3.1 Ω sq^−1^), which indicated that the P‐K‐*x* film had high conductivity and was tightly connected with CC substrate. Additionally, the hydrophilic property of the P‐K‐2.5 film was also confirmed by the contact angle measurements and compared with CC (Figure S1, Supporting Information). The contact angle of CC was about 131°, illustrating it is hydrophobicity. However, the fast disappearance of the water droplet on the P‐K‐2.5 film implied that P‐K‐2.5 possessed a good hydrophilic property. This phenomenon indicated that the P‐K‐2.5 contained oxygen‐/nitrogen functional groups, which was verified by the following energy‐dispersive X‐ray spectroscopy (EDX) and X‐ray photoelectron spectroscopy (XPS) results. For supercapacitor, the hydrophilic property of electrode material can promote deep the electrolyte wetting the surface and pore wall of the nanoscale carbon and make more inner surface ions accessible.

**Figure 2:**
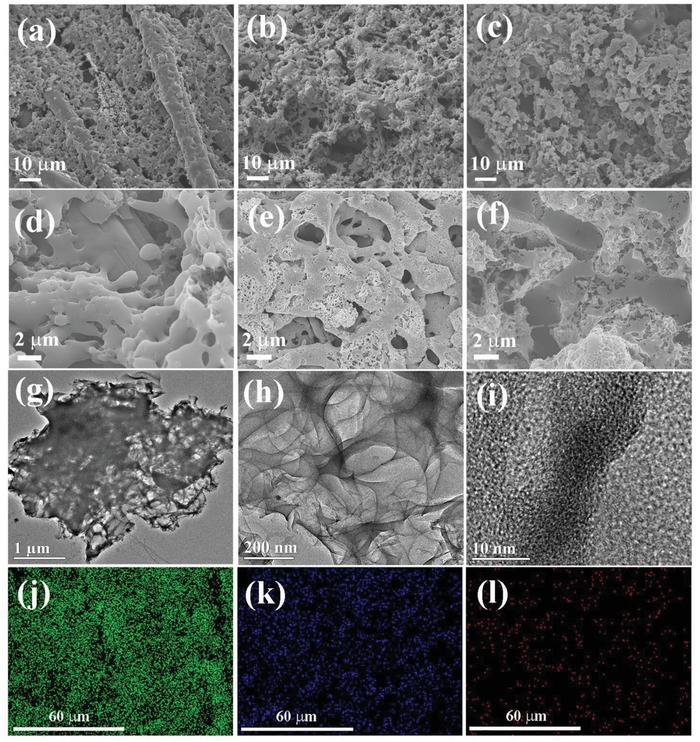
SEM of a,d) P‐K‐1.5, b,e) P‐K‐2.5, and c,f) P‐K‐3.5. g,h) TEM, i) HRTEM images, and elemental mapping images of j) C, k) O, and l) N of P‐K‐2.5.

**Figure** **2**a presents the scanning electron microscopy (SEM) images of the P‐K‐1.5. The prepared P‐K‐1.5 carbon film with some pores was evenly coated on CC. At the enlarged magnification (Figure 2d), the carbon surface was smooth. When the weight ratio of KOH and PIn was 2.5, meaningfully, a fishnet‐like porous P‐K‐2.5 film on CC was formed, which consisted of numerous well‐interconnected fibers (Figure 2b), and microscopically, the carbon surface contained abundant pore structures (Figure 2e). This was because that the high KOH amount can not only promote effectively the cracking, etching, and carbonization of polymers,^26^, ^27^ but also create the more pores by the released gases and structural transformation during the carbonization of polymers. However, further increasing the weight ratio of KOH and PIn to 3.5, it was found that the P‐K‐3.5 carbon film was mainly consisting of some clusters of irregular particles (Figure 2c,f). This indicated that the excessive KOH amount was adverse to the formation of well‐defined carbon film.

TEM images further reveal the microstructure of P‐K‐2.5 specimens (Figure 2g,h). The almost transparent region in TEM images was indicative of pore structures. The diameters of pore were between 100 and 200 nm. Additionally, it can be seen that P‐K‐2.5 specimens consisted of interconnected thin nanosheets. As seen from the high resolution transmission electron microscope (HRTEM) image (Figure 2i), lots of white spots reflecting micropores were observed, and they were evenly distributed across the material, suggesting that a primarily microporous structure with highly structural disorder was obtained after the activation of KOH. The elemental mapping results indicated that C, O, and N elements existed in the P‐K‐2.5 specimens and were distributed uniformly (Figure 2j,l).

**Figure 3:**
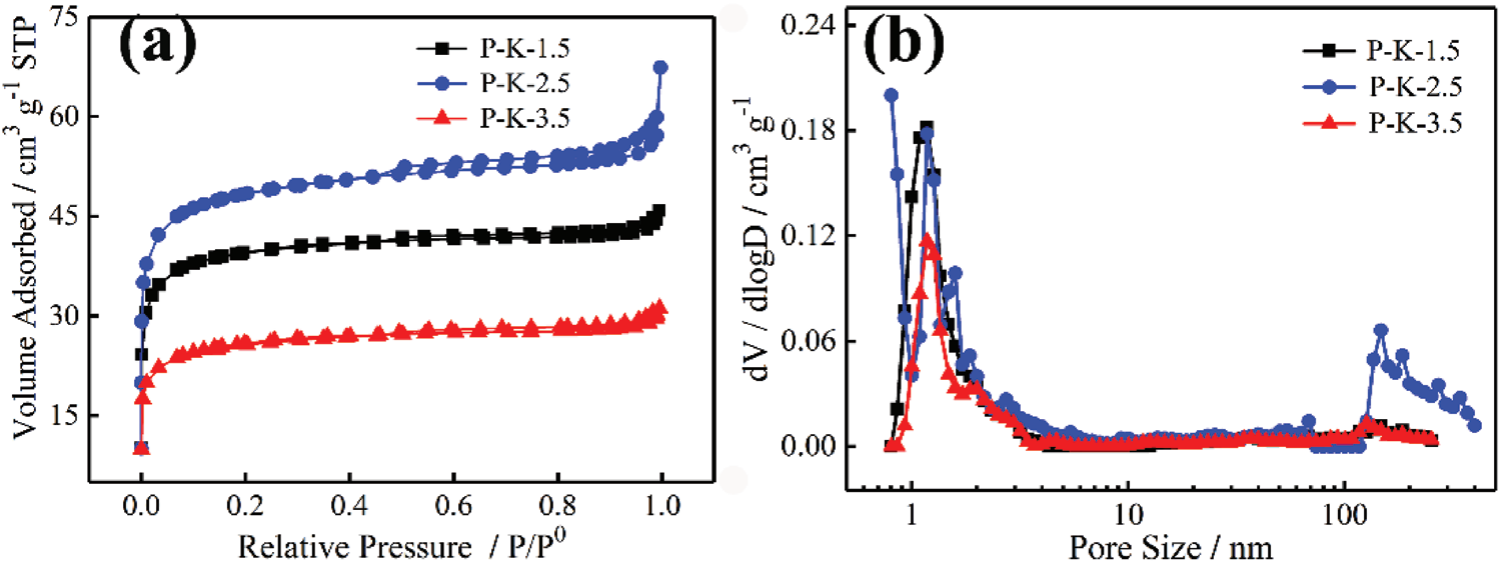
a) N_2_ adsorption–desorption isotherms and b) pore size distribution of P‐K‐*x*/CC electrodes.

**Figure** **3**a shows the nitrogen adsorption–desorption isotherms of P‐K‐*x*/CC electrodes. The P‐K‐1.5/CC and P‐K‐3.5/CC electrodes showed typical I isotherm,^22^ the steep increase in the adsorbed volume at a very low relative pressure (*P*/*P* ^0^ < 0.05) was indicative of the presence of abundant micropores. The P‐K‐2.5/CC electrode exhibited the combined characteristics of type I and IV isotherms, it presented a narrow knee at a very low relative pressure (*P*/*P* ^0^ < 0.05), indicating the existence of a well‐developed micro porosity, while the abrupt increase in the amount of adsorbed N_2_ at higher relative pressures approaching to 1.0 was attributed to the N_2_ adsorption in large sized mesopores or macropores up to hundreds of nanometers. Pore structure was a very important factor to determine the capacitance performance of carbon electrodes. Figure 3b shows the pore size distribution curves of P‐K‐*x*/CC electrodes. The pore size of P‐K‐1.5/CC and P‐K‐3.5/CC mainly focused on 1–2 nm. However, the P‐K‐2.5/CC not only showed abundant micropores and micropores with 1–3 nm but also had some macropores with 100 nm above. The pore structure parameters of P‐K‐1.5/CC, P‐K‐2.5/CC, and P‐K‐3.5/CC were summarized in **Table** **1**. It has been reported that the more than 0.5 nm pores were electroactively available in aqueous electrolytes for supercapacitors.^28^ Thus, the pore size of the P‐K‐*x* films benefited the aggregation of electrolyte ion in pores, and thereby decreased the resistance of electrolyte ion diffusion. Moreover, with increasing of the ratio of KOH and PIn from 1.5 to 3.5, the surface area and pore volume showed parabola trend and their maximum values were obtained as the ratio was 2.5, that is, the Brunauer–Emmett–Teller (BET) specific surface area (*S*_BET_) of P‐K‐1.5/CC, P‐K‐2.5/CC, and P‐K‐3.5/CC were 152, 185, and 98 m^2^ g^−1^, along with the pore volume of 0.071, 0.104, and 0.048 cm^3^ g^−1^, in which the law of influence of KOH amount on *S*_BET_ and pore volume of carbon materials was in accordance with our previous reported results.^29^ Note that the *S*_BET_ was far less than our previous reported values,^29^ mainly because the CC with *S*_BET_ of 3.5 m^2^ g^−1^ was dominant mass in the tested electrodes (Figure S2, Supporting Information).

**Table 1:** Pore parameters of P‐K‐1.5/CC, P‐K‐2.5/CC, and P‐K‐3.5/CC electrodes

Samples	*S*_BET_ [m^2^ g^−1^]	*D* [nm]	*V* [cc g^−1^]	Composition^a)^			% of total N1s		
				C%	O%	N%	N‐5	N‐6	N‐Q
P‐K‐1.5/CC	152	1.86	0.071	71.1	24	4.9	67.0	14.0	19.0
P‐K‐2.5/CC	185	2.26	0.104	78.6	17.6	3.8	63.9	11.3	24.8
P‐K‐3.5/CC	98	1.96	0.048	83.7	13.8	2.5	54.2	5.4	40.4

*S*_BET_: specific surface area. D: Adsorption average pore diameter. *V*: total pore volume

^a)^ Atom percent of elements obtained from XPS analysis

**Figure 4:**
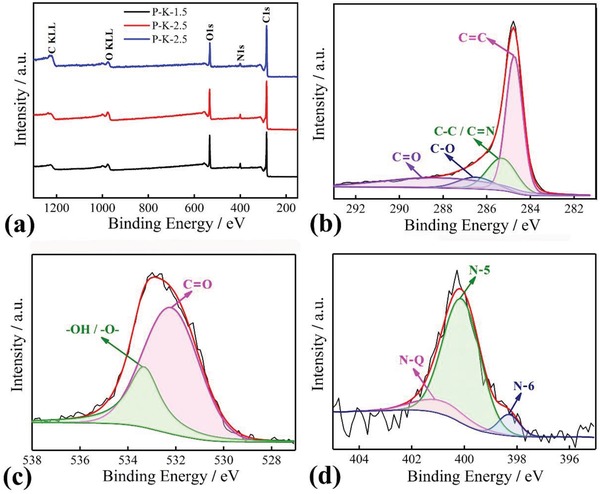
Wide‐scan XPS spectra of a) three samples, b) C1s, c) O1s, and d) N1s of P‐K‐2.5.

**Figure** **4**a shows wide‐scan XPS spectra of three samples. For the three samples, C, O, and N elements were determined, and their contents were listed in Table 1. With increasing of KOH amount, the contents of N and O elements deceased due to the aromatization and dehydrogenation was accelerated during the carbonization process. In comparison with O element, the content of N was more sensitive to the employed KOH amount during the process of activation, decreasing from 4.9 at% for P‐K‐1.5, to 3.8 at% for P‐K‐2.5, and 2.5 at% for P‐K‐3.5. Figure 4b–d shows the XPS spectra of C1s, O1s, and N1s of P‐K‐2.5. In Figure 4b, the chemical states of C1s were deconvoluted into four individuals component peaks corresponding to C—C (284.6 eV), C—N (285.4 eV), C—O (286.6 eV), and C=O (288.7 eV).^30^ The presence of C—N peak further proved that the nitrogen was successfully doped into the lattice of carbon film. In Figure 4c, the O1s spectrum revealed the existence of two oxygen‐based groups including OH or —O— (533.1 eV) or C=O (531.7 eV).^31^ In Figure 4d, three peaks at 398.4, 400.1, and 401.4 eV were assigned to pyridinic nitrogen (N‐6), pyrrolic nitrogen (N‐5), and quaternary nitrogen (N‐Q), respectively.^19^, ^32^ This result exhibited that the N atoms in indole units could be converted into N‐6, N‐5, and N‐Q in the process of the carbonization. As seen from Table 1, the N‐6 and N‐5 were transformed into N‐Q structure with the KOH amount increasing, which greatly improved the electron transport of carbon materials. For P‐K‐1.5 film and for P‐K‐2.5 film, the total contents of N‐5 and N‐6 were, respectively, higher than those of P‐K‐3.5 film and the reported carbons derived from PPy and PANI.^33^, ^34^, ^35^, ^36^, ^37^, ^38^ It was well known that the higher N‐5 and/or N‐6 content helped the capacitance enhancement of carbon materials through the contribution of their pseudocapacitance. Therefore, the PIn‐derived carbon materials would show superior capacitance relative to PPy and PANI‐derived carbons.

**Figure 5:**
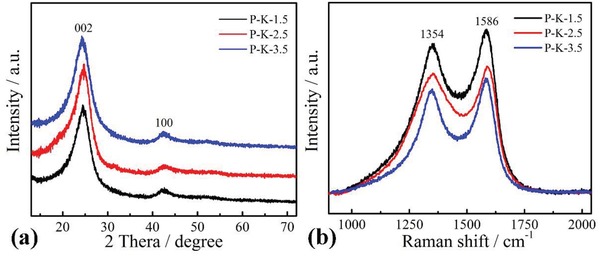
a) XRD patterns and b) Raman spectra of the samples.

**Figure** **5**a shows the XRD patterns of the prepared samples. There were two obvious diffraction peaks at 24° and 42.5°, respectively, corresponding to the (002) and (100) crystal planes of graphitic carbon, which confirmed the formation of amorphous carbon by carbonization treatment. Though the different KOH concentration treatment, the width and position of the (002) and (100) diffraction peaks remained unchanged, indicating KOH concentration did not seem to affect the crystal planes of carbon. According to Bragg's law: 2*d*sinθ = *nλ*, where *n* = 1, λ is the wavelength of incident wave (1.54178 Å),^39^, ^40^ the interlayer distance (*d*_(002)_) of graphitic layers was calculated to 0.371 nm, higher than that of bulk graphite (0.335 nm).^41^ The larger interlayer space was beneficial for the transfer of hydrated ions such as H^+^ (0.28 nm) and Na^+^ (0.358 nm) in the channels,^42^ which will enhance the energy storage capacity.

Raman spectra further provide the information about the characteristic G and D bands of the carbon materials. Figure 5b shows the Raman spectra of the prepared samples. Two characteristic peaks at 1354 and 1586 cm^−1^ corresponded to D‐band and G‐band, respectively. The D‐band is associated with *A*_1_ *_g_* symmetry assigned to local defects and disorder carbon with sp^3^ bonding, while the G‐band is related to the bond stretching of sp^2^‐hybridized carbon atoms in rings and chains according to the zone center *E*_2_ *_g_* mode.^43^, ^44^ Moreover, the higher intensity of G‐band than that of the D‐band indicated their partial graphitization.^45^ The integrated intensity ratios (*I*_D_/*I*_G_) of the P‐K‐1.5, P‐K‐2.5, and P‐K‐3.5 were 0.91, 0.93, and 0.90, respectively, indicative of a higher disorder degree of P‐K‐2.5.

**Figure 6:**
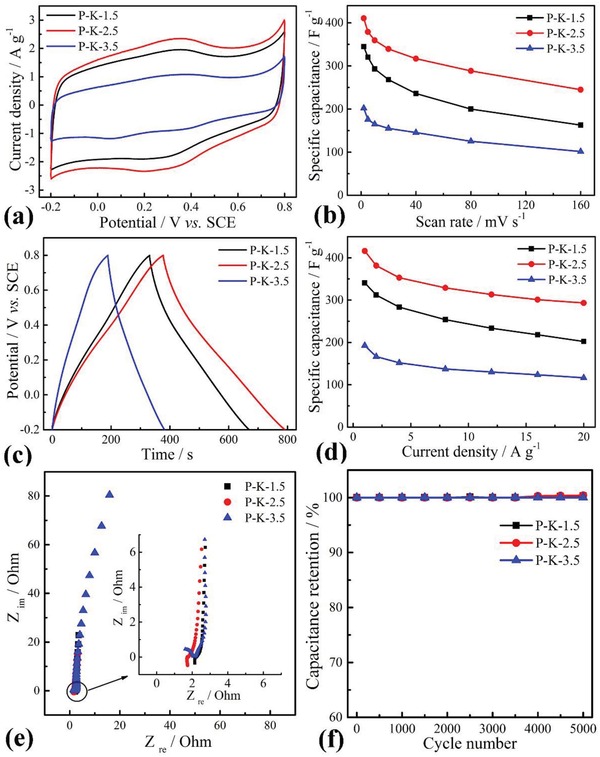
Electrochemical performances of P‐K‐1.5, P‐K‐2.5, and P‐K‐3.5 electrodes in 1.0 m H_2_SO_4_ solution. a) CVs at 5 mV s^−1^, b) specific capacitance at different scan rates, c) GCD at 1.0 A g^−1^, d) specific capacitance at different current densities, e) Nyquist plots, and f) cycling stability at 20 A g^−1^.

The electrochemical performances of P‐K‐1.5, P‐K‐2.5, and P‐K‐3.5 were evaluated in 1.0 m H_2_SO_4_ aqueous solution, as shown in **Figure** **6**. Note that the electrochemical activity of carbon cloth was almost negligible compared to that of active material (Figure S3, Supporting Information). At 5 mV s^−1^, the cyclic voltammetry (CV) curves of all samples exhibited a quasi‐square shape with a pair of redox peaks at the range from 0.2 to 0.4 V (Figure 6a), indicative of the electrochemical capacitance including the pseudocapacitance and electrical double‐layer capacitance. The pseudocapacitance was originated from the reversible redox conversion of oxygen functional groups or different redox states of N‐5 and N‐6 nitrogen existed in carbon film.^23^ Compared with their CVs, the P‐K‐2.5 exhibited a larger CV area mainly due to P‐K‐2.5 had a bigger *S*_BET_ and pore volume. This also indicated that the KOH concentration had greatly affected on the capacitance of activated carbon materials, namely, with the ratio increase of KOH and PIn, the enclosed CV areas increased firstly and then decreased; finally the optimum ratio of KOH and PIn was about 2.5. The highest specific capacitance of P‐K‐2.5 electrode reached 410 F g^−1^ at 2 mV s^−1^, which was higher than those of P‐K‐1.5 (344 F g^−1^) and P‐K‐3.5 (202 F g^−1^) (Figure 6b). Additionally, the galvanostatic charge–discharge (GCD) curves of the P‐K‐2.5 electrode at 1.0 A g^−1^ showed a longer discharge time than other samples (Figure 6c), indicative of higher electrochemical capacitance in line with the results of CVs curves. With increasing the scan rates and current densities, the calculated specific capacitance decreased according to different CVs and GCD curves (Figure S4, Supporting Information). In Figure 6d, the specific capacitance of P‐K‐2.5 electrode was 416 F g^−1^ at 1.0 A g^−1^, which was higher than those of P‐K‐1.5 (341 F g^−1^), P‐K‐3.5 (193 F g^−1^) and other reported N‐doped carbon material (**Table** **2**). At 20 A g^−1^, the specific capacitance of P‐K‐2.5 decreased to 293 F g^−1^. The capacitance retention of P‐K‐2.5 reached about 70% when current density was increased from 1.0 to 20 A g^−1^, which was higher than those of P‐K‐1.5 (≈59%) and P‐K‐3.5 (≈60%). These results indicated that P‐K‐2.5 had a good rate capability. This was mainly due to the bigger pore volume of P‐K‐2.5 film and the small connection resistance between P‐K‐2.5 film and CC. The former provided more ion diffusion channels and short diffusion distance; the latter facilitated the fast transfer of electrons. Additionally, the CVs and GCD of P‐K‐2.5 with 9.2 mg cm^−2^ areal loading were tested (Figure S5, Supporting Information), and the specific capacitance was 391 F g^−1^ at 1.0 A g^−1^.

**Table 2:** Specific capacitance of N‐doped carbon materials derived from different carbonaceous precursors in the literature

Carbonaceous precursors	Electrolyte	Current density/Scan rate	Specific capacitance [F g^−1^]	Ref.
PANI/bacterial cellulose	1 m Na_2_SO_4_	2 mV s^−1^	296	19
PANI nanotubes	6 m KOH	0.1 A g^−1^	366	20
PANI nanowires	6 m KOH	0.1 A g^−1^	327	21
PPy	EMImBF_4_	0.1 A g^−1^	290	22
Hollow PANI spheres	6 m KOH	0.5 A g^−1^	213	26
Schiff‐base frameworks	1 m H_2_SO_4_	1.0 A g^−1^	≈300	28
PPy/bacterial cellulose	6 m KOH	1.0 A g^−1^	120	33
PPy Aerogels	1 m H_2_SO_4_	0.1 A g^−1^	156	34
Gutamic acid	2 m KOH	1.0 A g^−1^	330.6	41
Resorcinol/formaldehyde	1 m H_2_SO_4_	1.0 A g^−1^	388	46
Rod‐shaped PANI	6 m KOH	2 mV s^−1^	369	47
PPy nanotubes	1 m H_2_SO_4_	1 mA cm^−2^	228	48
PPy hollow spheres	6 m KOH	1.0 A g^−1^	232	49
PANI nanowire arrays	6 m KOH	1.0 A g^−1^	307	50
PAN @ PANI nanofibers	1 m H_2_SO_4_	0.5 A g^−1^	335	51
Schiff‐base/resin copolymer	6 m KOH	2.0 A g^−1^	362	52
PAN	1 m H_2_SO_4_	0.2 A g^−1^	270	53
PDAN	6 m KOH	1.0 A g^−1^	228	54
Melamine	1 m H_2_SO_4_	5 mV s^−1^	271	55
PIn	1 m H_2_SO_4_	1.0 A g^−1^	416	This work

Schiff‐base frameworks were obtained from polyquinoneimine and triformylphloroglucinol.

From Nyquist plot (Figure 6e), the charge transfer resistance (*R*_ct_) obtained from the diameter of the semicircle was 0.1 Ω for P‐K‐2.5 electrode, which was slightly below those of P‐K‐1.5 (0.15 Ω) and P‐K‐3.5 (0.25 Ω) electrodes. The cycling stabilities of the three electrodes were also investigated using GCD at 20 A g^−1^. After 5000 cycles, no capacitance loss was observed (Figure 6f), indicative of a good electrochemical cycling stability.

The very favorable performance of P‐K‐2.5 could be attributed to following aspects: 1) High‐content N‐5 and N‐6 nitrogen increasing the pseudocapacitance, 2) N‐Q located at both the center and the edges of the graphite layer increasing the electronic conductivity, 3) The fishnet‐like carbon films with big pore volume and *S*_BET_ facilitating more ion diffusion and providing more reaction area, 4) The good hydrophilic property facilitating the wettability of the interface between the electrode materials and the electrolyte, 5) The carbon films directly anchored on CC without any binders and conducting additives reducing the connection resistance and ensuring high electronic transport, and 6) The fishnet‐like structure avoiding the capacitance loss produced by the expansion/shrinkage during the continuous charging and discharging.

**Figure 7:**
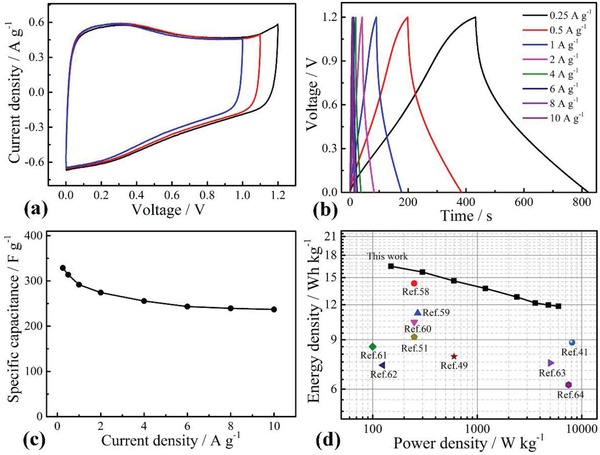
Electrochemical performances of button‐type supercapacitor based on P‐K‐2.5 electrodes using 1.0 m H_2_SO_4_ electrolytes. a) CVs with voltage window of 1.0, 1.1, and 1.2 V at 5 mV s^−1^, b) GCD at various current densities, c) specific capacitances at various current densities, and d) Ragone plot.

The capacitance performances of P‐K‐2.5 applied to the button‐type cell were also evaluated. **Figure** **7**a shows the CV with different voltage windows. It was demonstrated that the voltage window could be extended up to 1.2 from 1.0 V in 1.0 m H_2_SO_4_, similar to the results of other activated carbon.^56^ This reflected the nature of electrolytes and the structure stability of carbon materials. Beyond the voltage ranges, the side reactions such as electrolysis of water occur.^57^ The GCD curves at different current densities showed approximate symmetric patterns (Figure 7b). The low Coulombic efficiency was observed. The low Coulombic efficiency was mainly attributed to the charge leakage occurring during the discharging, which can decrease the time for discharging, or the water oxidation occurring on the film, which can increase the time for charging. However, this feature did not affect its specific capacitance. The corresponding specific capacitances at various current densities were also plotted in Figure 7c. It can be seen from Figure 7c that the specific capacitance of the single electrode decreased with the increase of the current density. From 0.25 to 10 A g^−1^, the specific capacitance decreased from 323 to 237 F g^−1^, the capacitance retention reached about 73%, indicative of high rate capability of cell. The Ragone plot was presented in Figure 7d, showing that the cell delivered a high energy density of 16.4 Wh kg^−1^ at a power density of 150 W kg^−1^, while the energy density remained at 11.9 Wh kg^−1^ at a higher power density of 6000 W kg^−1^. Figure 7d also compares the energy density of cell based on P‐K‐2.5 with other reported cells based on the heteroatom‐doped carbon using H_2_SO_4_ or KOH as electrolytes.^41^, ^49^, ^51^, ^58^, ^59^, ^60^, ^61^, ^62^, ^63^, ^64^ As seen, the cell based on P‐K‐2.5 exhibited superior energy density. Hence, the supercapacitor based on flexible P‐K‐2.5 films will have great potential for electronic applications.

**Figure 8:**
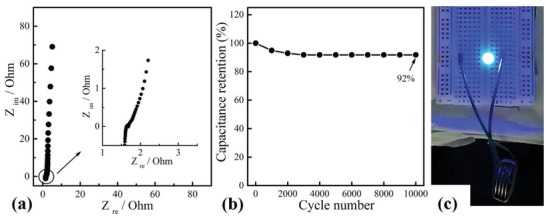
a) Nyquist plots, b) cycling stability at 10 A g^−1^, and c) a picture showing that four button‐type cells in serial can light a blue LED lamp.

**Figure** **8**a shows the Nyquist plot of cell. The plot included a semicircle and a sloping line at both high and low frequency regions respectively. The charge transfer resistance (*R*_ct_) obtained from the diameter of the semicircle was 0.2 Ω. The ESR was obtained from the *Z*_re_ axis intercept of the Nyquist plot, which was 1.7 Ω. The ESR value is a very significant parameter in surveying the maximum power density of a cell.^65^ The maximum power density of the cell was 67.4 kW kg^−1^ according to the equation *P*_max_ = *V* ^2^/4*RM*,^66^ where *V* is the voltage, R is the ESR, and *M* is the total mass of the electrode materials. The maximum power density value was mainly attributed to the design of porous binder‐free electrode, which greatly reduced the diffusion resistance between electrolytes and electrodes, and the contact resistance between electrode materials and current collectors. Moreover, the cell also exhibited good cyclic stability with 92% retention of initial capacitance after 10 000 cycles at 10 A g^−1^ (Figure 8b), indicating that the cell possessed a good electrochemical cycling stability. To testify the cell as an available power source, four cells were connected in series to assemble a power pack. As shown in Figure 8c, a blue light emitting diode (LED) lamp with a 3.0 V rated voltage was normally lighted up by the power pack, indicating that the P‐K‐2.5 electrode can function as a valuable carbon electrode material.

## 3 Conclusions

A binder‐free flexible FNPC/CC electrode was prepared by calcining the KOH‐activated polyindole physically coated on CC substrate. As‐prepared FNPC/CC electrode with 3.8 at% N content showed robust flexibility, mechanical behavior, good hydrophilicity, and abundant porosity. More significantly, the specific capacitance of FNPC reached 416 F g^−1^ at 1.0 A g^−1^ due to its high conductivity and tight connection with current collector without any binders and conducting additives. Furthermore, the cell based on FNPC/CC electrodes delivered high energy density of 16.4 Wh kg^−1^ at 150 W kg^−1^, maximum power density of 67.4 kW kg^−1^, and exhibited long‐term cycle stability of 92% of its initial capacitance after 10 000 cycles at 10 A g^−1^. This work not only puts forward a green, uncomplicated and low‐cost method to prepare fishnet‐like N‐doped porous carbon materials, but also gives a promising alternative for the application of carbon materials in supercapacitors.

## 4 Experimental Section

##### Materials

Indole (98%) was obtained from J & K Chemical Reagent Co., Ltd. Ammonium persulphate (APS, 98%) was obtained from Shanghai Vita Chemical Reagent Co., Ltd. Absolute ethyl alcohol, sulfuric acid (H_2_SO_4_, 98%), hydrochloric acid (HCl, 37%), were bought from Xilong Chemical Industry Incorporated Co., Ltd. Phosphoric acid (H_3_PO_4_, 85%), lithium perchlorate (LiClO_4_, 99%) and potassium hydroxide (KOH, 90%) were purchased from Aladdin Chemical Reagent Co., Ltd. CC (W1S1005) was purchased from Ce Tech Co. Ltd. Double deionized water was used in the experiment. All the chemicals were directly used without further purification.

##### Preparation of Materials

The synthesis process was described briefly as following: CC was cleaned ultrasonically in acetone, ethanol and deionized water separately for 30 min, then dried at 80 °C for 12 h. Then, PIn was obtained by chemical oxidative polymerization. In brief, 1.0 g indole monomer was dissolved in absolute ethanol (43 mL) at room temperature. Then 0.2 m APS aqueous solution (108 mL) was added drop by drop to indole solution with constant magnetic stirring condition. After 12 h, the precipitate was washed with double deionized water and ethanol, respectively, and dried at 85 °C in an oven for 24 h. Lastly, 2.0 g PIn and KOH were uniformly dispersed in 25 mL ethanol solution (50%) with continuous stirring for 8 h, and the mixture was coated on CC (12 mm × 10 mm). Subsequently, the PIn/KOH loaded CC was calcinated in tubular furnace at 700 °C under nitrogen atmosphere. After cooling, the product was thoroughly washed by 1.0 m HCl and distilled water until the pH of the water became neutral, then dried at 80 °C in an oven overnight, forming carbon electrode materials. The active materials on CC were 2 mg cm^−2^. All resulting carbon materials prepared by KOH activation were named as P‐K‐*x*, where *x* value shows the weight ratio of KOH and PIn.

##### Characterization

SEM and EDX were carried out by JSM‐6701F microscope. Transmission electron microscopy (TEM) images were obtained on FEI TECNAI G2 F20 microscope at an acceleration voltage of 200 kV. XPS was performed on the ThermoFisher K‐Alpha instrument. Raman spectra were performed on the Renishaw in Via 2000. Nitrogen adsorption–desorption measurement was performed on a Micromeritics ASAP 2020. The BET method was utilized to calculate the specific surface area. Electrochemical measurements of samples including CV, GCD, and electrochemical impedance spectroscopy measurements in 1.0 m H_2_SO_4_ electrolyte were performed with a CHI660E electrochemical workstation (Chen Hua Instruments Co., Shanghai, China), in which platinum wire and saturated calomel electrode (SCE) electrode were used as the counter and reference electrodes, respectively. A sealed symmetrical button‐type cell (CR2032) was assembled using 1.0 m H_2_SO_4_ as electrolyte, in which the diameter of round‐shape electrode was 1.0 cm. Specific capacitance was calculated showing in Supporting Information.

## Conflict of Interest

The authors declare no conflict of interest.

## Supporting information

Supporting Information

## References

[1] X. Yang , C. Cheng , Y. Wang , L. Qiu , D. Li , Science 2013, 341, 534. 23908233 10.1126/science.1239089

[2] F. Wang , X. Wu , X. Yuan , Z. Liu , Y. Zhang , L. Fu , Y. Zhu , Q. Zhou , Y. Wu , W. Huang , Chem. Soc. Rev. 2017, 46, 6816. 28868557 10.1039/c7cs00205j

[3] J. Huang , B. G. Sumpter , V. Meunier , Angew. Chem., Int. Ed. 2008, 47, 520. 10.1002/anie.200703864 18058966

[4] Q. Wang , J. Yan , Z. Fan , Energy Environ. Sci. 2016, 9, 729.

[5] Z. Wen , X. Wang , S. Mao , Z. Bo , H. Kim , S. Cui , G. Lu , X. Feng , J. Chen , Adv. Mater. 2012, 24, 5610. 22890786 10.1002/adma.201201920

[6] A. Stein , Z. Wang , M. A. Fierke , Adv. Mater. 2009, 21, 265.

[7] S. Kondrat , C. R. Pérez , V. Presser , Y. Gogotsi , A. A. Kornyshev , Energy Environ. Sci. 2012, 5, 6474.

[8] G. A. Ferrero , A. B. Fuertes , M. Sevilla , J. Mater. Chem. A 2015, 3, 2914.

[9] T. Lin , I. Chen , F. Liu , C. Yang , H. Bi , F. Xu , F. Huang , Science 2015, 350, 1508. 26680194 10.1126/science.aab3798

[10] L. Chen , Y. Lu , L. Yu , X. W. Lou , Energy Environ. Sci. 2017, 10, 1777.

[11] W. Jiang , D. Yu , Q. Zhang , K. Goh , L. Wei , Y. Yong , R. Jiang , J. Wei , Y. Chen , Adv. Funct. Mater. 2015, 25, 1063.

[12] A. Elmouwahidi , E. Bailón‐García , J. Castelo‐Quibén , A. F. Pérez‐Cadenas , F. J. Maldonado‐Hódar , F. Carrasco‐Marín , J. Mater. Chem. A 2018, 6, 633.

[13] R. R. Salunkhe , J. Tang , Y. Kamachi , T. Nakato , J. H. Kim , Y. Yamauchi , ACS Nano 2015, 9, 6288. 25978143 10.1021/acsnano.5b01790

[14] M. Li , J. Xue , J. Phys. Chem. C 2014, 118, 2507.

[15] S. Chen , J. Zhu , X. Wu , Q. Han , X. Wang , ACS Nano 2010, 4, 2822. 20384318 10.1021/nn901311t

[16] L. Zhang , X. Zhao , H. Ji , M. D. Stoller , L. Lai , S. Murali , S. Mcdonnell , B. Cleveger , R. M. Wallace , R. S. Ruoff , Energy Environ. Sci. 2012, 5, 9618.

[17] T. Lin , W. Lai , Q. Lü , Y. Yu , Electrochim. Acta 2015, 178, 517.

[18] Y. Liao , Y. Huang , D. Shu , Y. Zhong , J. Hao , C. He , J. Zhong , X. Song , Electrochim. Acta 2016, 194, 136.

[19] C. Long , D. Qi , T. Wei , J. Yan , L. Jiang , Z. Fan , Adv. Funct. Mater. 2014, 24, 3953.

[20] T. Zhu , J. Zhou , Z. Li , S. Li , W. Si , S. Zhuo , J. Mater. Chem. A 2014, 2, 12545.

[21] D. Yuan , T. Zhou , S. Zhou , W. Zou , S. Mo , N. Xia , Electrochem. Commun. 2011, 13, 242.

[22] L. Wei , M. Sevilla , A. B. Fuertes , R. Mokaya , G. Yushin , Adv. Funct. Mater. 2012, 22, 827.

[23] M. Wang , L. D. Duong , N. T. Mai , S. Kim , Y. Kim , H. Seo , Y. C. Kim , W. Jang , Y. Lee , J. Suhr , J. Nam , ACS Appl. Mater. Interfaces 2015, 7, 1348. 25545033 10.1021/am507656q

[24] C. Wu , S. Zhang , W. Wu , Z. Xi , C. Zhou , X. Wang , Y. Deng , Y. Bai , G. Liu , X. Zhang , X. Li , Y. Luo , D. Chen , Carbon 2019, 150, 311.

[25] D. Shen , G. Zou , L. Liu , W. Zhao , A. Wu , W. W. Duley , Y. N. Zhou . ACS Appl. Mater. Interfaces 2018, 10, 5404. 29357228 10.1021/acsami.7b14410

[26] J. Han , G. Xu , B. Ding , J. Pan , H. Dou , D. R. MacFarlane , J. Mater. Chem. A 2014, 2, 5352.

[27] B. Gupta , D. S. Chauhan , R. Prakash , Mater. Chem. Phys. 2010, 120, 625.

[28] M. Zhou , X. Li , H. Zhao , J. Wang , Y. Zhao , F. Ge , Z. Cai , J. Mater. Chem. A 2018, 6, 1621.

[29] J. Wu , W. Zhou , F. Jiang , Y. Chang , Q. Zhou , D. Li , G. Ye , C. Li , G. Nie , J. Xu , T. Li , Y. Du , ACS Appl. Energy Mater. 2018, 1, 4572.

[30] B. Wang , S. Li , X. Wu , J. Liu , J. Chen , J. Mater. Chem. A 2016, 4, 11789.

[31] S. Lei , L. Chen , W. Zhou , P. Deng , Y. Liu , L. Fei , W. Lu , Y. Xiao , B. Cheng , J. Power Sources 2018, 379, 74.

[32] W. Fan , Y. Xia , W. Tjiu , P. K. Pallathadka , C. He , T. Liu , J. Power Sources 2013, 243, 973.

[33] W. Lei , L. Han , C. Xuan , R. Lin , H. Liu , H. L. Xin , D. Wang , Electrochim. Acta 2016, 210, 130.

[34] Y. Sun , Z. Sui , X. Li , P. Xiao , Z. Wei , B. Han , ACS Appl. Nano Mater. 2018, 1, 609.

[35] J. Zhu , Y. Xu , Y. Zhang , T. Feng , J. Wang , S. Mao , L. Xiong , Carbon 2016, 107, 638.

[36] Y. He , X. Han , Y. Du , B. Song , P. Xu , B. Zhang , ACS Appl. Mater. Interfaces 2016, 8, 3601. 26479459 10.1021/acsami.5b07865

[37] W. Deng , Y. Zhang , Y. Tan , M. Ma , J. Electroanal. Chem. 2017, 787, 103.

[38] X. Li , Z. Sui , Y. Sun , P. Xiao , X. Wang , B. Han , Microporous Mesoporous Mater. 2018, 257, 85.

[39] Y. Li , B. Shen , X. Pei , Y. Zhang , D. Yi , W. Zhai , L. Zhang , X. Wei , W. Zheng , Carbon 2016, 100, 375.

[40] J. D. Rodriguez‐Blanco , S. Shaw , L. G. Benning , Nanoscale 2011, 3, 265. 21069231 10.1039/c0nr00589d

[41] G. Ma , Z. Zhang , H. Peng , K. Sun , F. Ran , Z. Lei , J. Solid State Electrochem. 2016, 20, 1613.

[42] A. G. Volkov , S. Paula , D. W. Deamer , Bioelectrochem. Bioenerg. 1997, 42, 153.

[43] Y. Gao , L. Li , P. Tan , L. Liu , Z. Zhang , Chin. Sci. Bull. 2010, 55, 3978.

[44] A. C. Ferrari , D. M. Basko , Nat. Nanotechnol. 2013, 8, 235. 23552117 10.1038/nnano.2013.46

[45] M. Wang , Y. Yang , Z. Yang , L. Gu , Q. Chen , Y. Yu , Adv. Sci. 2017, 4, 1600468. 10.1002/advs.201600468 PMC5396155 28435779

[46] N. P. Wickramaratne , J. Xu , M. Wang , L. Zhu , L. Dai , M. Jaroniec , Chem. Mater. 2014, 26, 2820.

[47] Q. Wang , J. Yan , Y. Xiao , T. Wei , Z. Fan , M. Zhang , X. Jing , Electrochim. Acta 2013, 114, 165.

[48] D. P. Dubal , N. R. Chodankar , Z. Caban‐Huertas , F. Wolfart , M. Vidotti , R. Holze , C. D. Lokhande , P. Gomez‐Romero , J. Power Sources 2016, 308, 158.

[49] B. Lv , P. Li , Y. Liu , S. Lin , B. Gao , B. Lin , Appl. Surf. Sci. 2018, 437, 169.

[50] G. Xu , H. Dou , X. Geng , J. Han , L. Chen , H. Zhu , Chem. Eng. J. 2017, 308, 222.

[51] F. Miao , C. Shao , X. Li , K. Wang , Y. Liu , J. Mater. Chem. A 2016, 4, 4180.

[52] D. Xue , D. Zhu , M. Liu , H. Duan , L. Li , X. Chai , Z. Wang , Y. Lv , W. Xiong , L. Gan , ACS Appl. Nano Mater. 2018, 1, 4998.

[53] Y. Shu , J. Maruyama , S. Iwasaki , S. Maruyama , Y. Shen , H. Uyama , RSC Adv. 2017, 7, 43172.

[54] D. Zhu , Y. Wang , L. Gan , M. Liu , K. Cheng , Y. Zhao , X. Deng , D. Sun , Electrochim. Acta 2015, 158, 166.

[55] U. B. Nasini , V. G. Bairi , S. K. Ramasahayam , S. E. Bourdo , T. Viswanathan , A. U. Shaikh , J. Power Sources 2014, 250, 257.

[56] X. Yang , Y. He , G. Jiang , X. Liao , Z. Ma , Electrochem. Commun. 2011, 13, 1166.

[57] M. A. Bissett , I. A. Kinloch , R. A. W. Dryfe , ACS Appl. Mater. Interfaces 2015, 7, 17388. 26196223 10.1021/acsami.5b04672

[58] Y. Li , G. Zhu , H. Huang , M. Xu , T. Lu , L. Pan , J. Mater. Chem. A 2019, 7, 9040.

[59] Y. Wang , Z. Chang , Z. Zhang , J. Lin , M. Qian , P. Wang , T. Lin , F. Huang , ACS Appl. Mater. Interfaces 2019, 11, 5999. 30648842 10.1021/acsami.8b19071

[60] P. Wang , W. Luo , N. Guo , L. Wang , D. Jia , Z. Zhao , S. Zhang , M. Xu , Chem. Phys. Lett. 2019, 730, 32.

[61] F. Liu , Z. Wang , H. Zhang , L. Jin , X. Chu , B. Gu , H. Huang , W. Yang , Carbon 2019, 149, 105.

[62] L. Zhou , H. Cao , S. Zhu , L. Hou , C. Yuan , Green Chem. 2015, 17, 2373.

[63] Z. Mao , C. Wang , Q. Shan , M. Wang , Y. Zhang , W. Ding , S. Chen , L. Li , J. Li , Z. Wei , J. Mater. Chem. A 2018, 6, 8868.

[64] W. Qian , J. Zhu , Y. Zhang , X. Wu , F. Yan , Small 2015, 11, 4959. 26150228 10.1002/smll.201500859

[65] Q. Cheng , J. Tang , J. Ma , H. Zhang , N. Shinya , L. Qin , Phys. Chem. Chem. Phys. 2011, 13, 17615. 21887427 10.1039/c1cp21910c

[66] S. Y. Wang , R. A. W. Dryfe , J. Mater. Chem. A 2013, 1, 5279.

