# Supplementary material for: Fishnet‐Like, Nitrogen‐Doped Carbon Films Directly Anchored on Carbon Cloths as Binder‐Free Electrodes for High‐Performance Supercapacitor
Source: Glob Chall. 2020 Jan 8;4(3):1900086. doi: 10.1002/gch2.201900086 (PMC7050067; doi:10.1002/gch2.201900086)
Supplement: Supplementary file 1 — Supporting Information [file GCH2-4-1900086-s001.pdf]

Copyright WILEY-VCH Verlag GmbH & Co. KGaA, 69469 Weinheim, Germany, 2020.

Supporting Information

for *Global Challenges,* DOI: 10.1002/gch2.201900086

Fishnet-Like, Nitrogen-Doped Carbon Films Directly Anchored on Carbon Cloths as Binder-Free Electrodes for High-Performance Supercapacitor

Jing Wu, Liming Xu, Weiqiang Zhou, Fengxing Jiang, Peipei Liu, Hui Zhang, Qinglin Jiang, and Jingkun Xu**

Supplementary Information

Fishnet-like Nitrogen-doped Carbon Films Directly Anchored on Carbon Cloths as Binder-free Electrodes for High-performance Supercapacitor

Jing Wu[a, ‖], Liming Xu[a, ‖], Weiqiang Zhou[a, *], Fengxing Jiang[a], Peipei Liu[a], Hui Zhang[a], Qinglin Jiang[a], Jingkun Xu[a, b, *]

[a] *Jiangxi Engineering Laboratory of Waterborne Coatings, Jiangxi Science and Technology Normal University, Nanchang 330013, China*

[b] *College of Chemistry and Molecular Engineering, Qingdao University of Science & Technology, Qingdao 266042, China*

‖ Jing Wu and Liming Xu contributed equally to this work.

* Corresponding author: E-mail: zhouwqh@163.com; xujingkun@tsinghua.org.cn

1 Calculation

1.1Three electrode

Specific capacitance values (*C*) of the material were calculated from cyclic voltammograms by means of Equation (1):**1**

$$C = \frac{\int_{E_1}^{E_2} i(E)d(E)}{2vm(E_2 - E_1)} \qquad (1)$$

Where E_1 and E_2 are the cutoff potentials in cyclic voltammetry, i(E) is the instantaneous current, i(E)dE is the total voltammetric charge obtained by integration of the positive and negative sweeps in the cyclic voltammograms, v is the scan rate, and m is the mass of the individual sample.

The specific capacitance were calculated from GCD curves by means of Equation (2):**2**

$$C = I \times \Delta t / (m \times \Delta V) \quad (2)$$

Where I and Δt are the discharge current and time respectively, ΔV is the scan rate, and m is the mass of the individual sample.

1.2 Two electrode

Specific capacitance of the material were calculated from cyclic voltammograms by means of Equation (3):3

$$C = \frac{4\int_{E_1}^{E_2} i(E)d(E)}{2vm(E_2 - E_1)} \quad (3)$$

Where E_1 and E_2 are the cutoff potentials in cyclic voltammetry, i(E) is the instantaneous current, i(E)dE is the total voltammetric charge obtained by integration of the positive and negative sweeps in the cyclic voltammograms, v is the scan rate, and m is the total mass of the active material at the two electrode.

The specific capacitance of the single electrode was calculated from GCD curves by means of Equation (4):4

$$C = 4I\Delta t / m\Delta V \quad (4)$$

Where I and Δt are the discharge current and time, respectively, ΔV is the voltage window, and m is the total mass of the active material at the two electrode.

The energy density (Wh kg^{-1}) and power density (W kg^{-1}) in the Ragone plot can be obtained from the GCD curves of the devices according to the Eqs: $E = 1/8 \times 1000/3600 \times C_{S2} \times \Delta V^2$ and $P = 3600 \times E/\Delta t$ (Cs_2: specific capacitance of the single electrode in devices, ΔV: the voltage window, Δt: the discharge time).4

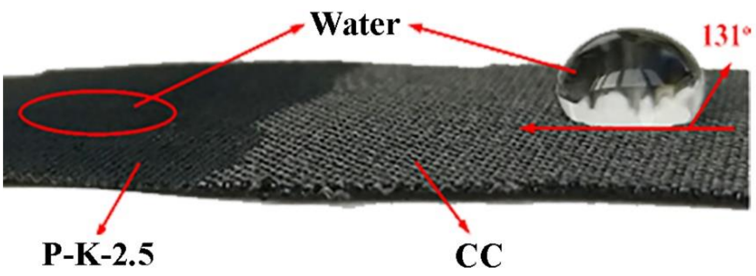

Figure S1 Contact angle of CF and P-K-2.5.

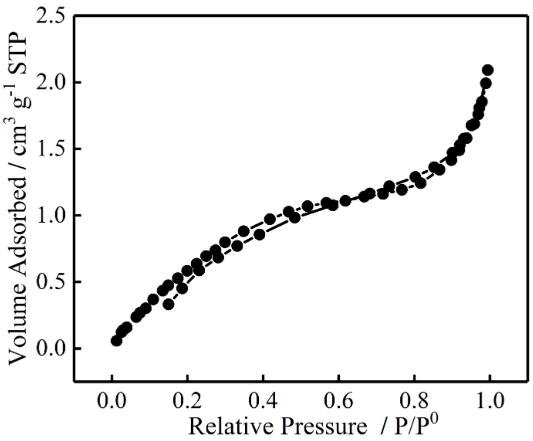

Figure S2. N_2 adsorption-desorption isotherms of CF.

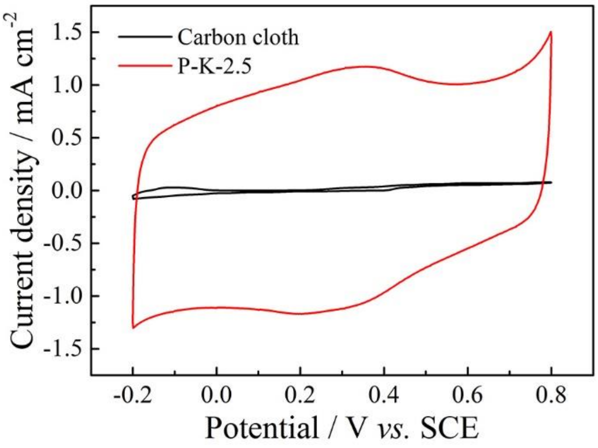

Figure S3. CVs of P-K-2.5 and carbon cloth electrodes in 1.0 M H_2SO_4 solution at 5 mV s^{-1}.

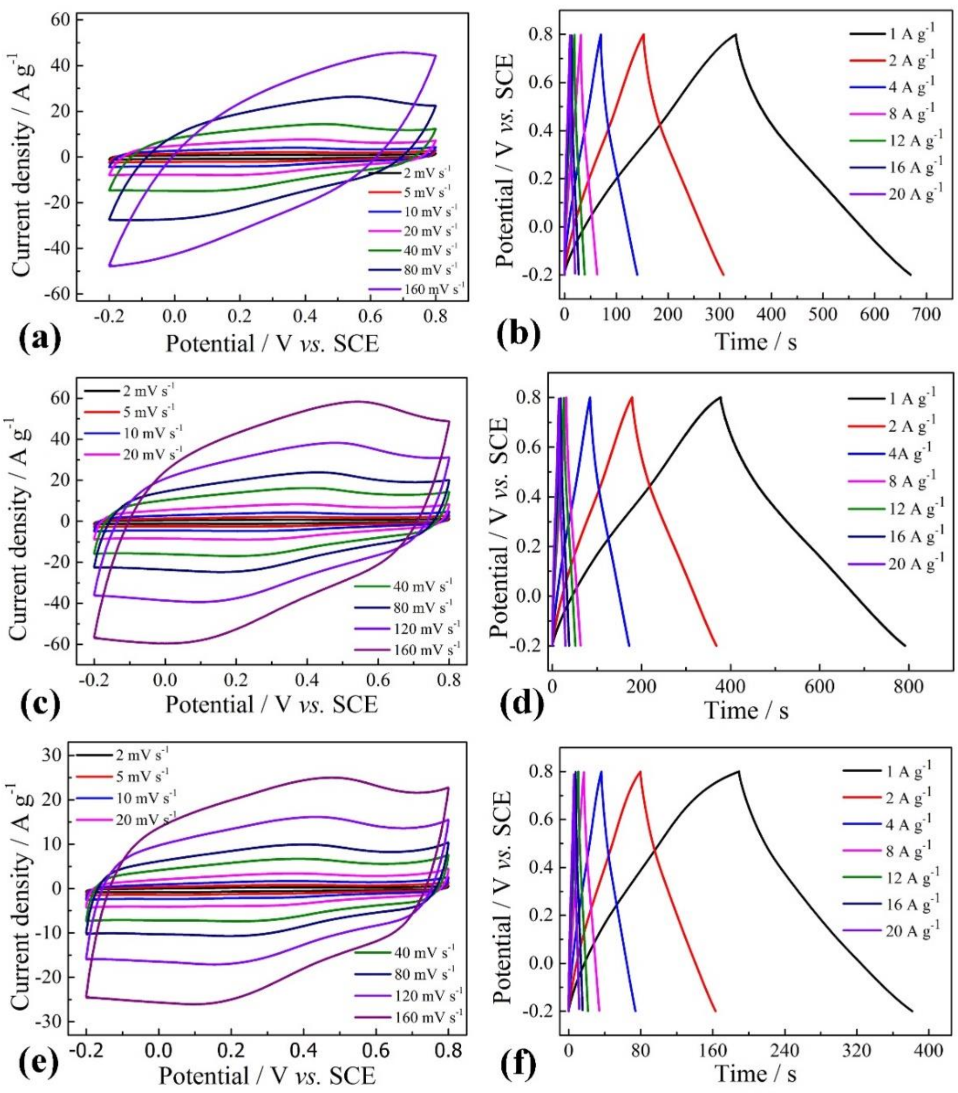

Figure S4. Electrochemical performance of P-K-1.5 (a, b), P-K-2.5 (c, d) and P-K-3.5 (e, f) in 1.0 M H_2SO_4.

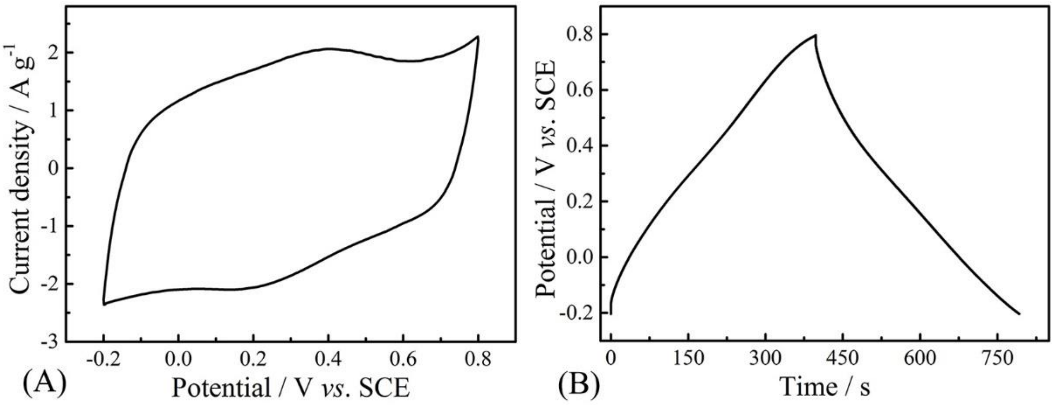

Figure S5. CVs and GCD of P-K-2.5 with 9.2 mg/cm^2 areal loading in 1.0 M H_2SO_4 solution at 5 mV s^{-1} and 1.0 A g^{-1}, respectively.

References

1 M. Plonska-Brzezinska, M. Lewandowski, M. Błaszyk, A. Molina-Ontoria, T. Luciński, L. Echegoyen, *ChemPhysChem* 2012, **13**, 4134.

2 C. Wu, J. Cai, Y. Zhu. Kaili Zhang, *RSC Adv.* 2016, **6**, 63905.

3 Z. Weng, Y. Su, D. Wang, F. Li, J. Du, Hu. Cheng, *Adv. Energy Mater.* 2011, **1**, 917–922

4 L. Hao, J. Ning, B. Luo, B. Wang, Y. B. Zhang, Z. H. Tang, J. Yang, A. Thomas, L. J. Zhi, *J. Am. Chem. Soc.* 2015, **137**, 219.
